# Antibody Titer Against Varicella Zoster Virus and Recombinant Varicella Zoster Vaccine in Hemodialysis Patients: What We Know, What We Should Know

**DOI:** 10.3390/life15040621

**Published:** 2025-04-07

**Authors:** Francesca K. Martino, Lucia F. Stefanelli, Martina Cacciapuoti, Elisabetta Bettin, Giuseppe Scaparrotta, Laura Gobbi, Dorella Del Prete, Lorenzo A. Calò, Federico Nalesso

**Affiliations:** Nephrology, Dialysis, Transplantation Unit, Department of Medicine (DIMED), University-Hospital of Padua, 35128 Padova, Italy; luciafederica.stefanelli@unipd.it (L.F.S.); martina.cacciapuoti@studenti.unipd.it (M.C.); elisabetta.bettin.1@studenti.unipd.it (E.B.); giuseppe.scaparrotta@aopd.veneto.it (G.S.); laura.gobbi_01@aopd.veneto.it (L.G.); dorella.delprete@unipd.it (D.D.P.); renzcalo@unipd.it (L.A.C.)

**Keywords:** varicella zoster virus, recombinant vaccination, antibody titer, hemodialysis

## Abstract

Background: Varicella zoster virus (VZV) infection can be life-threatening for fragile and immunosuppressed patients. Recombinant VZ vaccination (RVZV) has been recommended for vulnerable patients to reduce the risk of reactivation. Hemodialysis (HD) patients often have weakened immune systems and a high prevalence of comorbidities, which may justify the use of RVZV. This study examines the difference in VZ antibody levels following RVZV and its significance in HD patients. Methods: We measured the levels of immunoglobulin G antibodies against VZ (VZ-IgG) in the HD population. We also collected demographic and clinical data for each patient, including their age, length of time on dialysis, Charlson Comorbidity Index (CCI), and markers of nutritional and inflammatory status. Results: A total of 160 patients were evaluated, with 111 (69.4%) male and 143 (89.3%) Caucasian. The mean VZ-IgG levels after one year were significantly higher in patients who received RVZV than those who did not (2177 ± 834 versus 1494 ± 882, *p* < 0.001). Additionally, among all other risk factors, only CCI harmed the VZ-IgG levels in non-vaccinated HD patients (B −403 with 95%CI −778 −27.9, *p* = 0.039). Overall, 98.8% of patients were found to be seropositive for VZ, with only one patient in each group (RVZV and non-RVZV) testing negative. Conclusions: Patients who received RVZV showed higher VZ IgG levels after one year compared to those who did not. Moreover, unvaccinated patients with more comorbidities had lower anti-VZ IgG titers.

## 1. Introduction

Shingles is caused by the reactivation of latent VZV following a primary infection with VZV, and it presents as a unilateral, vesicular rash in a single dermatome accompanied by pain, which can be severe and persistent. The rate of shingles in the general population increases with age; the incidence is estimated to be between 6 and 8/1000 person-years over 60 and between 8 and 12/1000 person-years over 80 years of age [1]. It occurs due to a decline in VZ-specific immunity. A typical long-term issue is post-herpetic neuralgia [2,3], which is intense burning pain in one area that lasts for at least three months [4]. This pain is often resistant to treatment, affecting patients’ quality of life [5]. 

Hemodialysis (HD) patients have chronic low-grade inflammation, which compromises the immune response [6,7,8,9], significantly increasing their vulnerability to infections and the reactivation of dormant viruses [7]. Previous studies indicate that these patients face a twofold risk of viral reactivation compared to the general population [10,11] and a heightened likelihood of encountering severe complications associated with such events. Additionally, these individuals have higher resistance to antiviral treatment, necessitating longer treatment durations [12]. Furthermore, they are at a greater risk for neurotoxicity related to antiviral treatment [13]. Given these peculiarities, implementing a prophylactic strategy to prevent the reactivation of the varicella zoster virus (VZ) in this at-risk population seems a prudent action.

Since 2017 in the United States and 2018 in Europe, a recombinant vaccine for VZ (RVZV) has been available for adult patients over 50 and for those over 18 with immunological impairment to reduce the risk of VZ reactivation. According to its FDA and EMA approval [14,15], RVZV can prevent shingles [16,17] and post-herpetic neuralgia for at least four years and can reduce the rate of serious complications such as viral dissemination, stroke, encephalitis, and visual impairment without the risks of a live attenuated vaccine. The indications for fragile patients were determined in two studies on hematological patients [15]. One study showed a 68% reduction in VZ reactivation in people who had received an autologous stem cell transplant, and the other research observed an 87% reduction in patients with blood cancer. Afterward, other trials reported the potential usefulness in other immunological impairment conditions, such as in human immunodeficiency virus infection [18], in autologous stem cell transplantation [19], in kidney transplantation [20], in patients with solid tumors before or during chemotherapy [21], and in patients with immune-mediated diseases [22]. Conversely, only one recent report has described surrogate biomarkers for the efficacy of RVZV with one year of follow-up in hemodialysis patients [23]. No other study has explored the actual role of RVZV in shingle prevention in HD patients. Additionally, no studies have examined the baseline conditions and fragility to assess the risk of shingles among HD patients. 

Both humoral and cell-mediated immunity are involved in primary VZ infection and vaccination [24], but, while cell-mediated immunity is predominant in VZ reactivation [25,26], the role of humoral immunity is minor. Specifically, the only study published about the immune response to VZ in dialysis patients suggested that cellular and humoral immunity showed no impairment and seemed similar to that of matched healthy controls [27], revealing that the underlying mechanism is not entirely understood and needs further investigation. Conversely, some studies have shown a significant correlation between the VZ antibody titer and cell-mediated immunity tests [28,29,30,31,32] and a significant VZ antibody titer increase after vaccination [33,34,35]. Cell-mediated immunity assessment requires the analysis of qualitative/quantitative lymphocyte subpopulations and antigen-specific T-cells, a methodology that is currently not always feasible in all clinical contexts. In contrast, the VZ antibody titer is a widely used measure in evaluating VZ contact, as it is more straightforward, quicker, and cheaper to apply. In this clinical context, evaluating VZ IgG titers seems a reasonable strategy to understand the immune status versus VZ reactivation [36].

The VZ vaccination campaign for patients undergoing HD impacts public and private health system expenditures, which could represent a barrier to wide vaccine diffusion. For example, the RVZV vaccine costs approximately EUR 180 per person for a single dose in Italy. Given the estimated 45,000 dialysis patients in the country [37], the total financial burden related to this vaccination initiative would amount to around EUR 16 million. Despite the cost-effectiveness of VZ vaccination, which seems to favor its widespread diffusion, especially in vulnerable patients, definitive conclusions about the real benefit of RVZV are still lacking [38]. Further investigations into the role of the patient’s age, the vaccine’s price and efficacy, and the incidence of shingles complications are necessary. Considering the lack of cost-effectiveness evidence in the HD population, the uncertainties surrounding the vaccine’s efficacy for post-herpetic neuralgia [17], and the different rates of major complications between patients and treatment modalities [39], we aim to share our clinical experiences with RVZV vaccination to enhance the current understanding of its effects on HD patients. This retrospective report seeks to thoroughly discuss antibody titers against varicella zoster virus (VZ) as a potential indicator of the risk of developing shingles in patients undergoing hemodialysis (HD). Additionally, the study explores various clinical conditions that may influence the VZ-IgG levels in these patients to discover higher-risk patients. By identifying these factors, we aim to enhance the understanding of the shingles risk in HD patients and suggest the prioritization of RVZV administration among individuals on renal replacement therapy.

## 2. Materials and Methods

We conducted a retrospective study at Padua University Hospital in Italy to assess the antibody titer in HD patients and the impact of the clinical basal conditions on its value. The study was approved by the local ethics committee and conducted according to the principles expressed in the Declaration of Helsinki. 

All HD patients >19 years old were considered eligible for the study. 

For each patient, we evaluated the following features.

- Demographic characteristics such as age, gender, dialytic vintage, and history of shingles in the year before the blood examination were documented, According to the reports in clinical notes.
- Previous RVZV administration. Specifically, vaccinated patients received a two-dose series from August 2022 to January 2023, with a period between the first and second dose of about two months.
- The Charlson Comorbidity Index (CCI) was recorded to assess the patient’s fragility [40]. Specifically, the CCI adjusted by the patient’s age (+1 in patients between 50 and 59 years old, +2 in patients between 60 and 69 years old, +3 in patients between 70 and 79 years old, +4 in patients over 80 years old) was determined, evaluating the following conditions: diabetes, congestive heart failure, peripheral vascular disease, chronic pulmonary disease, liver disease, hemiplegia, renal disease, hematological or metastatic cancer, and acquired immunodeficiency syndrome.
- Blood examinations related to the patient’s well-being, such as hemoglobin, albumin, C-reactive protein (CRP), white cell count, platelet count, and urea, were evaluated. Anti-VZ IgG was detected. A serum sample was collected from each participant and tested by chemiluminescence using LIAISON^®^ VZV IgG, a semi-quantitative method performed with a standardized commercial kit (Diasorin Italia S.p.A, Saluggia, Italy). According to the manufacturer’s instructions, an anti-VZ IgG titer > 150 mIU/mL was scored positively.

We collected all blood samples during the regular monthly HD controls. Specifically, in RVZV patients, the test for VZ titers was performed one year after vaccination, while, in non-RVZV patients, we collected the sample in the first two months of 2024.

Sample size: As a retrospective study, we determined the sample size as 131 cases by applying the following equation for sample size calculation [41]:

n = [(r + 1)/r] × [σ^2^ × (Z_1−β_ + Z _1−α/2_)]/d^2^

where n = desired number of samples, Z_1−β_ = desired power (1.28 for 90% power), Z_1−α/2_ = standardized value for the corresponding confidence level (at a 99% CI or 1% type I error, it is 2.58), d = margin of error or rate of precision, and σ = standard deviation estimated according to the results published by Hielscher F on the hemodialysis population [18]. Furthermore, considering the size of our cohort, we decided to enroll all hemodialysis patients who consented to participate in the study to avoid selection bias. 

Statistical analysis: Continuous variables were reported as the mean ± standard deviation (SD) or median with interquartile range (IQR) according to their distribution, while categorical variables were reported as numbers or percentages. The Shapiro–Wilk test evaluated the normality of distribution for continuous variables. A paired *t*-test and the Mann–Whitney U-test were used to compare the levels of anti-VZ IgG between the groups according to the variable distribution. 

Univariate and multivariate linear regression were performed to identify the predictors of the anti-VZ IgG titer. All continuous variables that were non-normally distributed were normalized by natural log transformation. All reported *p*-values were two-sided, and statistical significance was set at *p* < 0.05. Statistical analysis was performed with IBM SPSS Statistics (Version 28.0).

## 3. Results

### 3.1. HD Cohort Description

A total of 160 HD patients were enrolled, of which 111 (69.3%) were male. Among them, 72 patients (45.3%) received two subsequent doses of RVZV within two months. In our patient cohort, the median age was 67.5 years, with a median Charlson Comorbidity Index (CCI) of 8. Notably, the mean anti-VZ IgG level was 1799 mIU/mL. The characteristics of the HD cohort are detailed in Table 1. 

### 3.2. Anti-VZ IgG Titer

We did not find any significant differences in the anti-VZ IgG levels among the explored variables based on gender, race, or the presence of diabetes and hypertension. In contrast, patients who received RVZV twelve months prior to blood sampling exhibited higher levels of anti-VZ IgG compared to non-vaccinated patients, with means of 2177 (±834) and 1494 (±882), respectively. Table 2 indicates that the differences in the anti-VZ IgG levels correlate with the categorical risk factors.

Additionally, the CCI showed a significant negative correlation with the anti-VZ IgG titer in non-vaccinated patients, while it exhibited no significant correlation in vaccinated patients. No significant correlation was found between anti-VZ IgG and the other continuous variables. Specifically, Table 3 presents the relationship between humoral immunity and the risk associated with the continuous variable factors.

**Figure 1:**
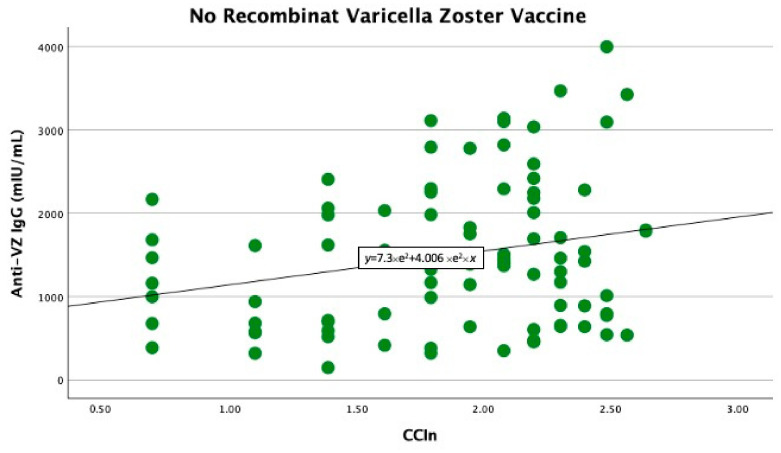
Significant linear relationship between anti-VZ IgG titer and CCI_n_ in non-vaccinated patients.

**Figure 2:**
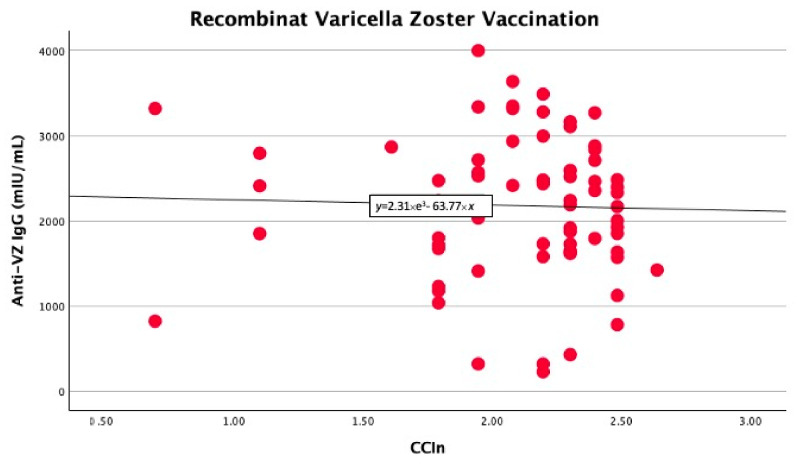
No significant linear correlation between anti-VZ IgG titer and CCI_n_ in vaccinated patients.

In the univariate linear regression analysis, we found that the CCI_n_ predicted significantly higher anti-VZ IgG titers only in non-vaccinated patients (B −403 with 95% CI −778 −27.9, *p* = 0.039). In contrast, in vaccinated patients, the comorbidity index did not significantly predict anti-VZ IgG titers (B −74.7 with 95% CI −571.9 and 422.4, *p* = 0.76), as shown in Figure 1 and Figure 2. 

Finally, in our cohort, only two patients (1.2%) had anti-VZ IgG levels below 150 mIU/mL; of these, one had received two doses of RVZV, while the other had no vaccination. Notably, among the HD patients who had received two previous doses of RVZV, one patient experienced a shingles episode in the year following vaccination.

## 4. Discussion

Our retrospective study revealed a remarkably high prevalence of HD patients with anti-VZ IgG titers exceeding 150 mUI/mL. Specifically, only two patients were VZ seronegative, one of whom had received RVZV in the year prior. Furthermore, we showed that patients who were vaccinated with RVZV in the previous year had significantly higher anti-VZ IgG titers compared to those who were not vaccinated. 

Finally, our report demonstrated a significant negative correlation between lower anti-VZ IgG titers and a higher comorbidity index in unvaccinated patients. Meanwhile, no other conditions significantly impacted the anti-VZ IgG levels.

### 4.1. Prevalence of Seropositive VZV 

The large number of HD patients testing positive for VZ antibodies, along with the patients’ ages, indicates that chickenpox was widespread in Italy during the twentieth century. The Italian population born before 1998 did not receive the chickenpox vaccine in childhood, according to the Italian National Immunization Plan prior to 2003. Therefore, the presence of anti-VZ antibodies in non-vaccinated individuals suggests a history of infection. After a long period following the primary infection, the well-preserved anti-VZ IgG titer is likely related to a more robust immunity response to VZ infection than chickenpox vaccination [33]. Notably, one non-vaccinated patient and one RVZV HD patient had undetectable anti-VZ IgG titers. The non-vaccinated patient may have never had a primary VZ infection, or their titer could have declined to undetectable levels. Conversely, the vaccinated patient demonstrated an inadequate response to RVZV, as did the patient who experienced shingles after two doses of RVZV. These findings in HD patients highlight the need to prioritize VZ vaccination for seronegative patients and those with lower antibody titers based on their childhood chickenpox history.

### 4.2. VZ Anti-IgG Titer After RVZV 

The higher levels of anti-VZ IgG titers in vaccinated patients underscore an immune response at one year after RVZV. Our findings are aligned with this and support the findings of previous studies, which showed a significant increase after vaccination [42,43,44] due to the effect of the immune response to the vaccination. Specifically, our report is consistent with a study by Hielscher F. et al. [23], which found a 2.2-fold increase in VZV-specific IgG levels and a significant correlation with neutralizing activity over one year. 

It is challenging to assess the effectiveness of RVZV in HD patients without studies focused on this specific population. Currently, no reports exist about the rate of VZ reactivation in HD patients who have received RVZV; the only reports on RVZV have evaluated the safety of the vaccination and its side effects [45], as well as humoral and cell-mediated immunity [23] during the first year after vaccination. However, given the aging of HD patients and their comorbidities, along with the lack of significant differences in the humoral and cellular immune responses to VZ [27] compared to the general population, we can speculate that HD patients may experience vaccine efficacy comparable to the lower end of the confidence interval for individuals over 70 years old. Therefore, HD patients might have vaccine efficacy of around 85% in preventing shingles, based on the findings of Cunningham et al., where the vaccine efficacy ranged from 84.2% to 93.7% in older patients [46]. Simultaneously, HD patients could have vaccine efficacy of about 70% for post-herpetic neuralgia, which was estimated to be between 68.7% and 97.1% in older adults [46]. Additional reports on the incidence of shingles and neuralgia following RVZV are necessary to determine the actual benefits of vaccination in dialysis patients, particularly in light of the questionable advantages of RVZV concerning post-herpetic neuralgia even within the general population [17]. Consequently, we plan to extend our study’s observation period to four years, emphasizing the ongoing nature of our research and the necessity for further investigation in this area. 

Given the uncertain evidence regarding vaccination’s benefits in all hemodialysis patients, assessing antibody titers prior to vaccination may be an appropriate procedure in clinical practice. This initial step could help to identify higher-risk patients, which is a significant aspect of personalized medicine. Due to their unique circumstances, dialysis patients present challenges in utilizing biomarkers to determine pathophysiological processes or prognosis [47,48,49,50,51]. This peculiarity arises not only from end-stage kidney disease but also from the potential effects of the hemodialysis procedure on biomarker levels [52,53,54,55,56]. In this context, the results of anti-VZ IgG assessment over time in dialysis patients could enhance our understanding of the kinetics of VZ antibodies, regardless of their immune system characteristics [6,7,8,57], thereby further emphasizing the importance of personalized medicine. Future studies are required to support these intriguing hypotheses.

### 4.3. Comorbidity Score and VZ Anti-IgG Titer

Interestingly, our series revealed a negative correlation between the CCI score and VZ anti-IgG titer in non-vaccinated patients, whereas vaccinated patients showed no significant correlation. Our findings align with other studies, indicating a decline in anti-VZ antibody titers among frail and older patients [58,59] and a heightened risk of VZ reactivation in high-comorbidity HD patients. The influence of comorbidities on the risk of shingles is well documented; other reports identify a high CCI as a risk factor for the development of shingles in the general population [60,61] and in specific groups, such as stroke patients [62]. Meanwhile, the lack of a significant correlation between VZ anti-IgG titers and the CCI in vaccinated patients suggests a robust humoral immune response to RVZV, highlighting its effectiveness even among frail patients. Based on these findings, stratifying the shingles risk by the CCI seems to be a reasonable strategy for the RVZV campaign in HD patients to prioritize vaccine administration. 

Our study has some limitations. The first are those associated with the retrospective study design and analysis. Secondly, the number of patients could limit the reliability of our analysis, reducing the actual effects of other covariates. Nevertheless, we enrolled all our HD patients and calculated the sample size according to the only previous report on hemodialysis patients. Thirdly, we did not consider the possible differences in antibody titers between the dialysis prescriptions. Still, the retrospective nature of our report and the high prevalence of bicarbonate dialysis in our cohort limited the reliability of this type of sub-analysis. 

## 5. Conclusions

Our study cohort showed a high prevalence of seropositivity for VZV among vaccinated and unvaccinated patients. Vaccinated patients exhibited significantly higher levels of anti-VZ IgG than unvaccinated patients, suggesting an effective immune response to vaccination regardless of the patient’s overall health status. Conversely, among unvaccinated patients, those with more comorbidities had lower anti-VZ IgG titers. This finding suggests that greater patient fragility may increase the risk of VZ reactivation. Consequently, a higher CCI and lower anti-VZ IgG titer could help the nephrologist to assess the RVZV priority in HD patients.

## Figures and Tables

**Table 1:** Characteristics of HD patients.

Variable	
Age ^ (years)	67.5 [55.2–77.5]
Dialytic vintage ^ (months)	33 [14–71]
Male °	111 (%)
Caucasian °	143 (91.1%)
Diabetes °	46 (28.7%)
Hypertension °	150 (93.8%)
Charlson Comorbidity Index ^	8 [6–10]
Albumin ^ (g/L)	37 [33–39]
Hemoglobin ^ (g/L)	107 [98–113]
White cell count ^ (n/mm^3^)	6180 [5030–7999]
Platelet count ^ (n/mm^3^)	180,000 [150,000–246,000]
C-reactive protein ^ (mg/L)	3.04 [1.09–7.9]
Urea ^ (mmol/L)	22.6 [19.6–27.4]
Anti-VZ IgG (mIU/mL) *	1799 (±923.7)

Notes: ° Categorical variables reported as number (perceptual value). * Normally distributed variables reported as mean (standard deviation). ^ Non-normally distributed variables reported as median [interquartile range].

**Table 2:** Anti-VZ IgG titers according to basal conditions.

Condition			*p*
Gender	Female	1826 (±922)	0.57
	Male	1736 (±933)	
Race	Caucasian	1777 (±922)	0.39
	Other	1982 (±947)	
Diabetes	Present	1826 (±922)	0.44
	Absent	1736 (±933)	
Hypertension	Present	1784 (±938)	0.43
	Absent	2022 (±679)	
RVZV	Administrated	2177 (±834)	<0.001
	No administrated	1494 (±882)	

Notes: RVZV, recombinant vaccine for varicella zoster.

**Table 3:** Pearson’s rho indices between continuous variables and anti-VZ titer.

	All HD Patients (n: 160)		Vaccinated HD Patients (72)		Non-Vaccinated HD Patients (88)	
Variable	Rho	*p*	Rho	*p*	Rho	*p*
Age	0.72	0.36	0.04	0.71	0.6	0.57
Dialytic vintage	0.76	0.34	0.10	0.41	0.39	0.71
CCI_n_	0.218	<0.001	−0.031	0.79	−0.245	0.02
Hemoglobinn	0.128	0.11	−0.66	0.58 0.77	0.038	0.2
Albumin	0.06	0.44	0.036		0.07	0.51
CRP	−0.32	0.69	0.047	0.69	−0.39	0.72
Urea	−0.12	0.12	0.011	0.93	−0.17	0.12

Notes: CCI_n_, Charlson Comorbidity Index Normalized.

## Data Availability

The data presented in this study are available on request from the corresponding authors.

## Abbreviations

The following abbreviations are used in this manuscript:

VZV	Varicella Zoster Virus
RVZV	Recombinant Varicella Zoster Vaccination
HD	Hemodialysis
VZ-IgG	Immunoglobulin G Antibodies against Varicella Zoster
CCI	Charlson Comorbidity Index
SD	Standard Deviation
IQR	Interquartile Range
